# Investigation of the role of rosmarinic acid treatment in regulating inflammation, cell damage, and angiogenesis in rat ovarian torsion and detorsion models^1^

**DOI:** 10.1590/s0102-865020200030000004

**Published:** 2020-05-20

**Authors:** Uğur Değer, Yunus Çavuş

**Affiliations:** I MD, Department of Obstetrics and Gynecology, Memorial Hospital, Diyarbakır, Turkey. Technical procedures, histopathological examinations, manuscript preparation and writing, final approval.

**Keywords:** Rosmarinic Acid, Caspase 3, Vascular Endothelial Growth Factor A, Tumor Necrosis Factor-alpha, Ovary, Rats

## Abstract

**Purpose:**

To investigate the protective effect of rosmarinic acid (RA) in ovarian ischemia/reperfusion injury using biochemical, histopathological, and immunohistochemical methods.

**Methods:**

Wistar female rats (n = 32) were randomly divided into four groups: control, ischemia, ischemia-reperfusion, and ischemia-reperfusion with RA. Rosmarinic acid was given at a dose of 50 mg/kg by oral gavage three hours after reperfusion. Malondialdehyde (MDA) levels and glutathione peroxidase (GSH-Px) activities were determined in the ovary tissue homogenates for each rat.

**Results:**

In the ischemia-reperfusion with RA group, the epithelial cells are regularly regulated at the periphery, and the degenerative changes in preantral and antral follicle cells are reduced. Follicle cells and cells in the corpus luteum showed a decrease in vascular endothelial growth factor (VEGF) expression, while VEGF demonstrated a positive reaction in vascular endothelial cells and stromal cells. The TNF-α expression due to the decreased degenerative effect and inflammation was positive in the macrophage cells. The expression of caspase-3 as an apoptosis change was negative in antral follicle cells and granular cells around the antral follicle.

**Conclusion:**

Different doses of RA may be useful in preventing ischemic damage after vascularization, inflammation, and apoptotic development after ischemia/reperfusion.

## Introduction

Ovarian torsion is a necrotic condition in the tissue that causes a significant decrease in the blood flow of the ovaries. Detorsion can cause significant damage to the tissue, even if the blood flow returns. Most cases of ovarian torsion occur during the reproductive period. Symptoms of ovarian torsion can be defined as acute abdominal pain with nausea, vomiting, and positive peritoneal findings. Adnexal cysts are thought to be risk factors for ovarian torsion in cases of ovarian growth, such as ovarian hyperstimulation or pregnancy, and the hypoxicity of ovarian propria and infundibulopelvic ligaments^1^.

In the reperfusion process, the production of excess reactive oxygen species (ROS) with increased neutrophil infiltration causes increased damage. Cell membrane lipids are the most sensitive structures affected by ROS, and several substances are produced during the lipid oxidation process. Malondialdehyde (MDA) is one of the most important end products of lipid peroxidation and is used to indicate the level of oxidative damage^2^.

Rosmarinic acid (RA) is a naturally occurring polyphenolic antioxidant found in numerous common herbal plants and is isolated from herbal balm mint plants, including *Melissa officinalis, Rosmarinus officinalis*, and *Prunella vulgaris* ^3-5^. Moreover, RA has demonstrated to have antioxidant, anticarcinogenic, anti-inflammatory, antidepressant, and antimicrobial effects^6-8^. Rosemary extracts have been used as an antioxidant to improve sperm quality and fertility^9^.

Caspases are a family of genes that maintain homeostasis through regulating apoptosis and inflammation. Yacobi *et al*.^10^ reported that gonadotropins play an important role in the prevention of pro-caspase-3, pro-caspase-7, and even caspase-3 and caspase-7 biosynthesis, and the addition of these hormones in vitro leads to a reduction in apoptosis.

Tumor necrosis factor-α (TNF-α) is one of the important mediators of inflammation, and it increases in the early stages of ischemia and reperfusion in the inflammatory state^11^. The TNF-α receptors are present on the cell membrane and are soluble in plasma. After reperfusion, neutrophil infiltration, and activation, cytokines, such as nitric oxide, TNF-α, and free oxygen radicals, are formed in the ovarian tissue.

The VEGF is a key regulator of both vasculogenesis and angiogenesis^12^ and is known as a neurotrophic and angiotrophic factor. Therefore, it induces the proliferation of endothelial cells and increases the permeability of the vessel wall^13,14^. Moreover, VEGF is closely associated with angiogenesis in the follicular phase and is important for nutritional support and the development of the corpus luteum and stroma^15^.

The aim of this study was to investigate the protective effect of RA in ovarian ischemia/reperfusion injuries using biochemical, histopathological, and immunohistochemical methods.

## Methods

### Experimental design and surgical procedure

All procedures performed in this experiment were approved by the Ethics Committee for the Treatment of Experimental Animals (Dicle University Faculty of Medicine, Turkey). Healthy Wistar female rats (250–280 g) were maintained under 22°C ± 1°C and 12 h light/dark cycles with ad libitum and free access to standard pellets. Their oestrus cycles were evaluated daily via a vaginal smear. Anesthesia was applied before the surgical procedure because of their high anxiety. Intramuscular ketamine hydrochloride (50 mg/kg Ketalar; Eczacıbaşı, Istanbul, Turkey) and xylazine hydrochloride (10 mg/kg Rompun; Bayer Türk İlaç Ltd, Istanbul, Turkey) were administered to each rat for this purpose.

In all of the groups, a midline abdominal incision of 2.5 cm (laparotomy) was performed under sterile conditions. After midline laparotomy, the left adnexa, including tubal and ovarian vessels, was rotated by 360^o^ in a clockwise direction. The rotated adnexa was fixed to the abdominal muscles with a 5/0 silk suture in the torsion and detorsion groups. The skin was sutured with 5/0 silk. After 2 hours of ischemia, the blood flow was re-allowed for 2.5 hours of reperfusion. For this procedure, animals in all groups were reanaesthetized and laparotomy was performed through the previous incision sites. Finally, rats’ blood samples were taken, and the ovaries were surgically removed after preparation of the adnexa.

Groups were randomly divided as follows:

- ***Control group (n = 8)*:** After anesthetizing all the experimental animals, their ovaries were surgically opened and then closed. The blood and ovarian tissues of the animals were taken.
- ***Ischemia group (n = 8)*:** The ovaries of the anesthetized animals were surgically opened, and the left ovaries were sealed to cause ischemia.
- ***Ischemia-reperfusion group (n = 8)*:** After 2 hours of ischemia, the blood flow was re-allowed for 2.5 hours of reperfusion. Then, the animals were sacrificed with an overdose of anesthetic, and the ovarian tissues were taken.
- ***Ischemia-reperfusion with RA (n = 8)*:** Rosmarinic acid was given at a dose of 50 mg/kg by oral gavage (according to T_max_) three hours after reperfusion.

Rosmarinic acid is water soluble, according to the literature, and the extraction efficiency of this compound in infusions is about 90%^16^. A dosage of about 1.6 mg / kg is the recommended dosage for an adult human weighing 70 kg. Therefore, it is possible to consume about 110 mg of RA per day. The low dose (10 mg/kg) used in the rats corresponds to the amount of RA that can be consumed by humans in the form of spices, herbal teas, and infusions. It considers the conversion factor of 6.17 resulting from the faster metabolism in rats^17^. It is indicated that rats can manage a high-dose (5 times) diet with a stronger therapeutic effect. In our study, 50 mg/kg of RA was applied^18^. There were no rat deaths in the experimental process of this study.

### Malondialdehyde and glutathione peroxidase assays

The MDA levels and glutathione peroxidase (GSH-Px) activities were determined in the ovary of each rat, and the average values of each group were calculated. Each ovary sample was prepared as a 10% homogenate (according to weight) in 0.9% saline using a homogenizer on ice. Then, the homogenate was centrifuged at 2000 rpm for 10 min, and the supernatant was collected. The MDA levels were determined using the double heating method of Draper and Hadley^19^. The GSH-Px activity was measured by the method of Paglia and Valentine^20^. An enzymatic reaction was initiated by the addition of hydrogen peroxide (H_2_O_2_) to a tube that contained reduced nicotinamide adenine dinucleotide phosphate, reduced glutathione, sodium azide, and glutathione reductase. The change in absorbance at 340 nm was monitored by spectrophotometry. Data were expressed as U/g of protein.

### Measurement of superoxide dismutase activity

The total superoxide dismutase (SOD) activity was determined with a SOD detection kit (RANSOD kit, Randox Co., UK) according to the manufacturer’s instructions. The SOD accelerates the conversion of the toxic superoxide (produced during the oxidative energy processes) to hydrogen peroxide and molecular oxygen. This method employs xanthine and xanthine oxidase to generate superoxide radicals that react with 2-(4-iodophenyl)-3-(4- nitrophenol)-5-phenyltetrazolium chloride (INT) to form a red formazan dye. The SOD activity is measured by the degree of inhibition of this reaction. One unit of SOD causes 50% inhibition of the rate of reduction of INT under the assay conditions. Absorbance measurements were taken at 505 nm, and the SOD levels were determined through a standard curve and expressed as U/mg of protein^21^.

### Measurement of catalase activity

The tissue catalase (CAT) activity was assayed spectrophotometrically by monitoring the decomposition of H_2_O_2_ using the procedure from Aebi^22^. Briefly, 0.5 mL of 30 mM H_2_O_2_ in 50 mM phosphate buffer (pH 7.0) was added to 1 mL of tissue supernatant (diluted 1:10), and the consumption of H_2_O_2_ was followed spectrophotometrically at 240 nm for 2 min at 25˚C. The molar extinction coefficient was 43.6 L/mol/cm for H_2_O_2_. The CAT activity was expressed as millimole of H_2_O_2_ consumed per min per milligram of tissue protein.

### Histopathological analysis

The ovarian samples were fixed with neutral buffered 10% formalin solution. After preservation, ovarian samples were directly dehydrated in a graded series of ethanol and embedded into paraffin wax. In addition, 5 mm sections were cut with a microtome (Rotatory Microtome, Leica, RM 2265, Germany) and mounted on the coated slides. The sections were stained with Masson’s Trichrome in order to be observed under a light microscope.

Masson’s Trichrome staining procedure was as follows:

1. Deparaffinize slides and hydrate to deionized water.
2. Mordant in preheated Bouin’s Solution at 56°C for 15 minutes or at room temperature overnight.
3. Cool slides in tap water (18–26°C) contained in a Coplin jar.
4. Wash in running tap water to remove yellow color from sections.
5. Stain in Working Weigert’s Iron Hematoxylin Solution for 5 minutes.
6. Wash in running tap water for 5 minutes.
7. Rinse in deionized water.
8. Stain in Biebrich Scarlet-Acid Fucshin for 5 minutes.
9. Rinse in deionized water.
10. Place slides in Working Phosphotungstic/Phosphomolybdic Acid Solution for 5 minutes.
11. Place slides in Aniline Blue Solution for 5 minutes.
12. Place slides in Acetic Acid, 1%, for 2 minutes. Discard solution.
13. Rinse slides, dehydrate through alcohol, clear in xylene and mount.

### Immunohistochemical staining

Samples were fixed with 10% formaldehyde solution, decalcified with 5% ethylene-diamine-tetraacetic acid (EDTA), dehydrated in a graded series of ethanol, and then embedded in paraffin wax. Then, 4–5 µm thick sections were cut with a microtome (Leica, Germany) and placed on coated slides. Sections were brought to distilled water and washed three times for 5 min in phosphate-buffered saline (PBS, pH 7.4) (catalogue # 10010023, Thermo Fisher Scientific, US). To unmask antigen sites, the slides were incubated with EDTA solution in a microwave for 110 minutes at 3 x 90^o^C. The sections were washed three times for 5 min in PBS and were incubated with hydrogen peroxide (catalogue # TA-015-HP, Thermo Fisher Scientific, US) for 20 min. Ultra V block (TA-125-UB, Thermo Fisher Scientific, US) was applied to the sections for 8 min prior to the addition of the primary antibodies, which were left on overnight in VEGF antibody (catalogue # ab1316, 1:100), TNF-α antibody (catalogue # ab6671, 1:100), and caspase-3 antibody (catalogue # ab4051, 1:100), all from Abcam, US. The sections were washed three times for 5 min in PBS and then were incubated with biotinylated secondary antibody (catalogue # TP-125-BN, Thermo Fisher Scientific, US) for 14 min. After washing with PBS, streptavidin peroxidase (catalogue # TS-125-HR, Thermo Fisher Scientific, US) was applied to the sections for 15 min. The sections were washed three times for 5 min in PBS. Diaminobenzidine (catalogue # TA-012-HDC, Thermo Fisher Scientific, US) was applied to sections for up to 20 min as a chromogen. Control slides were prepared using the same procedure, without primary antibodies. Counterstaining was done using Harris’s haematoxylin for 45 s, dehydrated through ascending alcohol series and cleared in xylene (Product Number: HHS32 Sigma, hematoxylin solution, Harris Modified, Sigma-Aldrich, 3050 Spruce Street, Saint Louis, MO, 63103, USA). Slides were mounted with Entellan® (lot: 107961, Sigma-Aldrich, St. Louis, MO, USA) and examined under a light microscope (Olympus, Germany).

### Statistical analysis

Statistical analyses of the histopathological and biochemical parameters were performed with SPSS (Version 22.0, SPSS Inc., Chicago, IL, USA). The descriptive statistics were presented as the median (min-max) and mean ± standard deviation values. Because the distribution of data was not normal, a nonparametric test was used (Kruskal–Wallis test). The significance of the difference between more than two groups was evaluated using the Kruskal–Wallis test because the data did not meet the assumptions of the parametric test for ANOVA. Subsequently, post-hoc tests with the Bonferroni correction Mann–Whitney U test were used to determine which groups differed with a pair-wise comparison.

## Results

### Biochemical findings

The statistical results of histopathological parameters are given in Table 1 in detail. We evaluated biochemical, histopathological, and immuno-histochemical parameters to determine the efficacy of RA on ischemia and reperfusion injuries in rat ovaries. The biochemical results are shown in Table 1 using the Kruskal–Wallis test. When all the groups were compared using the Kruskal–Wallis test, a significant difference was observed between the groups (*p* < 0.05; Table 1). In paired comparisons with the Bonferroni correction Mann–Whitney U test, significant results were observed in all groups (*p* < 0.0083; Table 2).

**Table 1:** Statistical comparison of biochemical results of groups using the Kruskal–Wallis test (*statistically significant result, *p*<0.05).

Groups		MDA	SOD	CAT	GSH
	Mean	6.12200	3.49575	.04350	365.54075
Control	*N*	8	8	8	8
	Std. Deviation	.534087	.373856	.005632	10.849007
Ischemia	Mean	9.12913	1.84738	.02263	303.39138
	*N*	8	8	8	8
	Std. Deviation	.517390	.292794	.006457	15.694564
Ischemia/Reperfusion	Mean	12.37063	1.68425	.01600	312.58838
	*N*	8	8	8	8
	Std. Deviation	1.062242	.127228	.002330	6.924180
Ischemia/Reperfusion + RA	Mean	6.30540	3.50650	.04250	365.53340
	*N*	10	10	10	10
	Std. Deviation	.278179	.195014	.003923	7.984131
Total	Mean	8.35376	2.68482	.03182	338.45582
	*N*	34	34	34	34
	Std. Deviation	2.629440	.915624	.013028	31.065566

		**MDA**	**SOD**	**CAT**	**GSH**

Chi-square		27.498	24.995	25.954	24.830
*df*		3	3	3	3
Asymp. Sig.		.000*	.000*	.000*	.000*

**Table 2:** Statistical comparison of the biochemical results of groups using the Bonferroni correction Mann–Whitney U test (comparison of biochemical results of parameters as a binary group) (1: control group; 2: ischemia group; 3: ischemia/reperfusion group; 4: ischemia/reperfusion + RA group; *p<0.0083 is statistically significant using the Bonferroni correction Mann–Whitney U test).

Groups	*P-value*			

	MDA	SOD	CAT	GSH
1-2	.0014*	.0012*	.0012*	.0016*
1-3	.0018*	.0025*	.0021*	.0028*
1-4	.0011*	.0017*	.0019*	.0014*
2-3	.0019*	.0024*	.0021*	.0022*
2-4	.0011*	.0017*	.0016*	.0022*
3-4	.0018*	.0012*	.0019*	.0029*

### Histological findings

**Figure 1:**
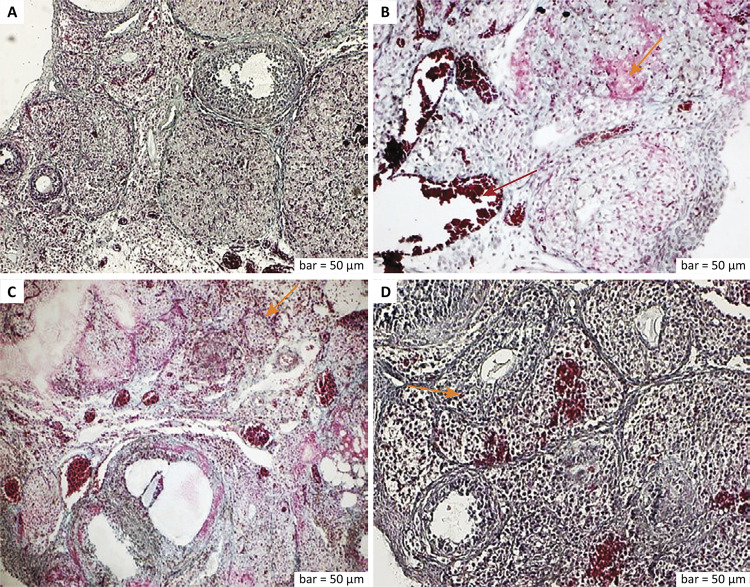
a. Masson’s Trichrome staining (control group). The germinal epithelium was regular and lined throughout the periphery. The cortical preantral and antral follicles were oval, and the luteal cells of the corpus luteum were full of secretory contents. Irregular connective tissue fibers, solitary-localized cells, regular blood vessels, and small hemorrhages were observed in the stromal area. b. Masson’s Trichrome staining (ischemia group). Degeneration of germinal epithelial cells, deterioration of preantral and antral follicles, and increased inflammation in the cortical area were observed (*yellow arrow*). Hyper-dilated and congestion in blood vessels (*red arrow*), edema around blood vessels, degenerative changes in collagen fibers were seen. c. Masson’s Trichrome staining (ischemia-reperfusion group). Degenerated preantral and antral follicles with decreased size, apoptotic follicle cells with pyknotic nuclei, increased inflammation outside the follicles (*yellow arrow*), and intensive congestion in blood vessels were observed. d. Masson’s Trichrome staining (Ischemia-reperfusion + RA group). Epithelial cells were regularly organized on the periphery, and decreased degenerative changes in the preantral and antral follicle cells were observed. Granular cells were small and regular, and some of them had dense secretory granules (*yellow arrow*). Around the follicles, the collagen fibers were arranged in parallel, and the connective tissue cells were solitarily distributed. Scale bar = 50 μm.

**Figure 2 f02:**
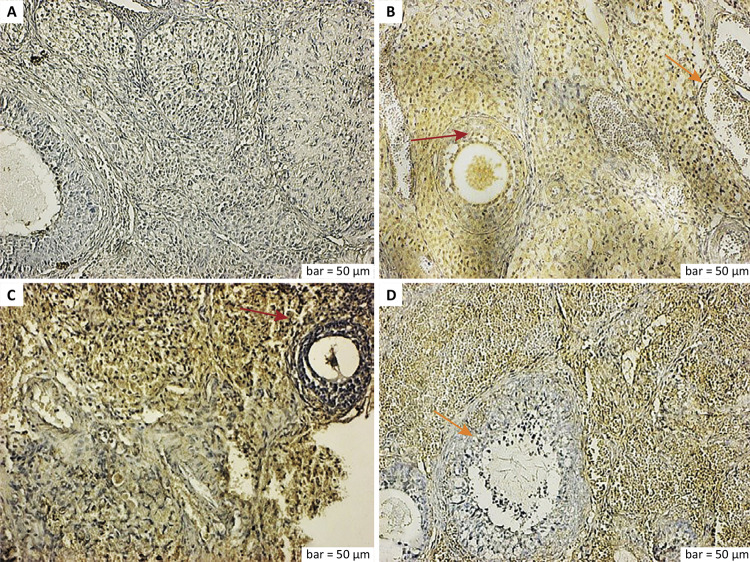
a. VEGF immunostaining (control group). The control group results showed negative expression of VEGF in vascular endothelial cells and stromal macrophage cells outside the preantral and antral follicles. b. VEGF immunostaining (ischemia group). Positive VEGF expression was seen in degenerative preantral and antral follicle cells (*red arrow*), dilated vascular endothelial cells, and dense inflammatory cells (*yellow arrow*). c. VEGF immunostaining (ischemia-reperfusion group). The VEGF expression was increased in the luteal cells of the corpus luteum and the vascular endothelial and inflammatory cells (*red arrow*). d. VEGF immunostaining (ischemia-reperfusion + RA group). Follicle cells and cells in the corpus luteum showed a decrease in VEGF expression (*yellow arrow*), while the VEGF expression was positive in vascular endothelial and stromal cells. Scale bar = 50 μm.

**Figure 3 f03:**
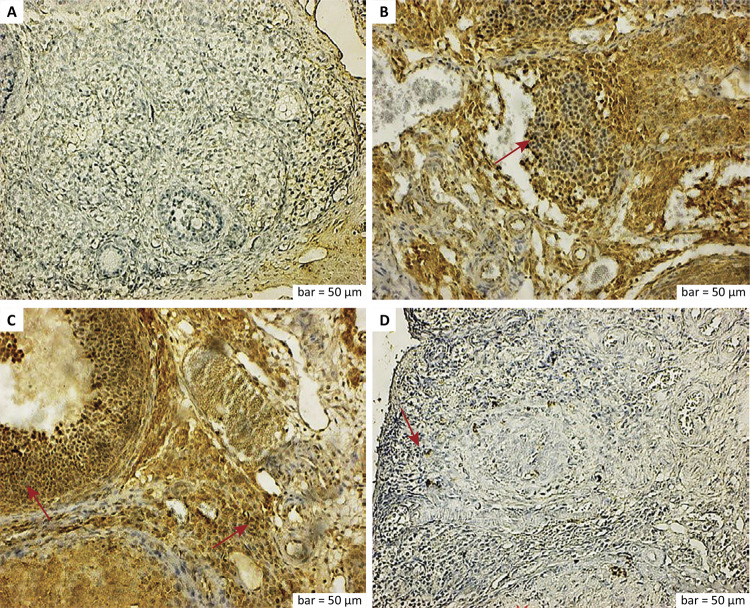
a. TNF-α immunostaining (control group). Preantral and antral follicle cells and stromal macrophage cells showed a negative TNF-α expression, whereas the TNF-α expression was positive in corpus albicans cells. b. TNF-α immunostaining (ischemia group). The TNF-α was positively expressed in the degenerative follicular cells and numerous inflammatory cells around the stromal blood vessels in the ischemia group (*red arrow*). c. TNF-α immunostaining (ischemia-reperfusion group). The TNF-α expression was positive in the granular cells of the antral follicles, vascular endothelial cells, and inflammatory cells (*red arrows*). d. TNF-α immunostaining (ischemia-reperfusion + RA group). The TNF-α expression was positive in granular cells, blood-vessel endothelial cells, and inflammatory cells in the antral follicles (*red arrow*). The TNF-α expression was positive in some macrophage cells distant from the preantral and antral follicles, while the TNF-α expression was negative in other areas. Scale bar = 50 μm.

**Figure 4 f04:**
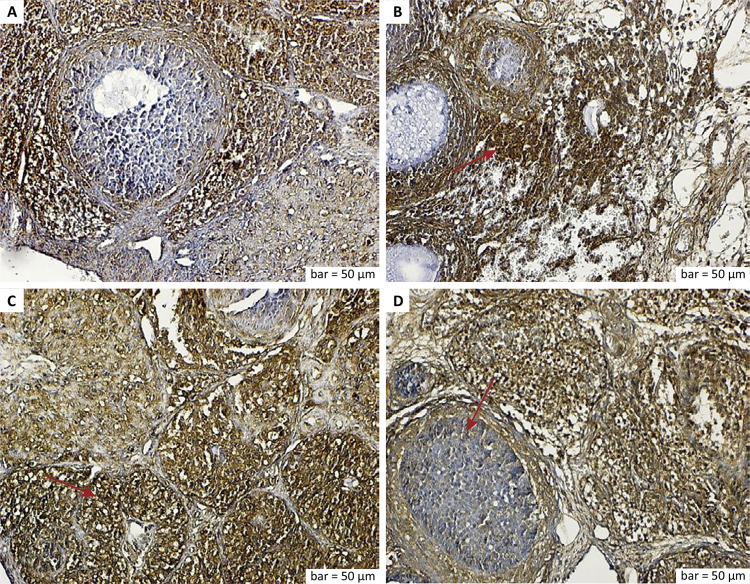
a. Caspase-3 immunostaining (control group). The control group caspase-3 results showed that caspase-3 expression was negative in germinal epithelial cells, granular cells of preantral and antral follicles, and endothelial cells. However, it was positive in the stromal cells around the follicle. b. Caspase-3 immunostaining (ischemia group). The positive expression of caspase-3 was observed in degenerated granular cells of preantral and antral follicles, luteal cells of the corpus luteum, and many inflammatory cells in the stromal region (*red arrow*). c. Caspase-3 immunostaining (ischemia-reperfusion group). Caspase-3 was positively expressed in granular cells in mature antral follicles and inflammatory cells in the stromal region (*red arrow*). d. Caspase-3 immunostaining (ischemia-reperfusion + RA group). Caspase-3 expression was negative in the preantral follicle cells and granular cells around the antral follicle (*red arrow*), whereas it was positive in some stromal cells and corpus luteum cells. Scale bar = 50 μm.

The histological and immunohistopathological findings of all groups are shown in detail below (Figs. 1 to 4 [Fig f02] [Fig f03] [Fig f04]).

## Discussion

Ovarian ischemia is a gynecological condition that causes significant damage from the torsion of the ovaries. Detorsion of the twisted tissue damages the ischemic ovaries and causes reperfusion damage. Therefore, the duration of the detorsion is considered to be increased with a conservative treatment option^23^. Such injuries are more dangerous in ischemic ovaries that do not receive antioxidant and anti-inflammatory treatment, which indicates the importance of conservative treatment^24^. Many pathological studies have described tissue damage in ovarian torsion and ovarian lipid peroxidation, inflammation, and cell death^25^. Detorsion involves the production of toxic oxygen species (ROS) by the return of the blood flow after ischemia^26^. Excessive production of ROS can cause a significant increase in lipid peroxidation. However, increased MDA and antioxidant defense systems of the human body have shown to cause damage^27^.

The enzymatic antioxidant defense system, which includes SOD, CAT, and GSH-Px, reacts to scavenge the free radicals to protect tissues from ischemia/reperfusion injury^28,29^. Ischemia stimulates chemotactic factors and leads to the migration of polymorphonuclear leukocytes to the ischemic region, which also generates ROS^30^. The increase in the MDA levels in tissue is considered a marker of tissue injury and indicates the level of lipid peroxidation in the tissue. Peroxidation of lipids disrupts the cell membrane structure. Lipid peroxidation reaction can indirectly be shown by MDA, which indicates cellular damage caused by ROS effects^29^. There is an antioxidant enzyme (SOD, CAT, and GSH-Px) defense against this oxidative stress in the ovary. Antioxidant enzymes that play critical roles in converting radicals into nonradical products in the antioxidant defense mechanism include SOD, CAT, and GSH-Px^29-31^. Changes in tissue antioxidant enzyme activities (CAT, SOD, and GSH-Px) and changes in the tissue levels of MDA in the ischemia/reperfusion group demonstrated a significant ischemia/reperfusion injury due to ovarian torsion. Our results have shown that the MDA levels were increased, and the SOD, CAT, and GSH-Px activities were decreased in ovarian torsion (Tables 1 and 2). Rosmarinic acid is a polyphenol group and has antioxidant, anti-inflammatory, and antimicrobial properties. It helps prevent cell damage caused by free radicals. Rosmarinic acid has shown to have anti-oxidative and anti-apoptotic effects against ischemia/reperfusion damage in brain tissue via signaling factor 2 and heme oxygenase-1 associated with nuclear erythroid factor 2^32^. Rosmarinic acid in ovariectomized rats was found to be effective in the oxidative damage parameters in the serum. It has been reported that MDA can reduce tissue-induced effects, such as lipid peroxidation and DNA damage^33^. Our results have shown that MDA levels increased, and SOD, CAT, and GSH-Px activities decreased in ovarian torsion (Tables 1 and 2).

Vascular endothelial growth factor (VEGF), a potent stimulator of endothelial cell proliferation and migration and a promoter of vascular permeability, is the major angiogenic factor that controls follicular angiogenesis^34^. Angiogenesis is one of the major features of the early corpus luteum. In addition, VEGF is the most important factor in the regulation of both normal and abnormal angiogenesis^35^. Expression of VEGF increased in the ischemia group in the degenerative preantral and antral follicle cells, dilated vascular endothelial cells, and dense inflammatory cells, and in the ischemia-reperfusion group in the corpus luteum luteal cells and vascular endothelial and inflammatory cells (Fig. 2 b,c). In the ischemia-reperfusion with RA group, the follicle cells and cells in the corpus luteum showed a decrease in VEGF expression, while a positive VEGF reaction was observed in the vascular endothelial and stromal cells (Fig. 2d).

Inflammation is another pathophysiological mechanism in ischemia/reperfusion injury, with excessive oxidative stress caused by reperfusion after ischemia. In patients with adnexal torsion, it is important to prevent or reduce ovarian tissue damage. It is thought that keeping the ischemia time short is essential. Early conservation surgery (ovarian loosening = detorsion) is considered the most effective clinical approach in the treatment of ovarian torsion in girls and adolescents^36^.

Adnexal torsion reduces blood flow and increases the lactic acid, hypoxanthine, and lipid peroxide levels. Although detorsion is performed for adnexal torsion as a treatment, it is followed by neutrophil infiltration and an increase in free oxygen radicals and cytokines, such as nitric oxide and TNFα^37^. In one study, there was an increase in the ovaries of rats exposed to ischemia/reperfusion in proinflammatory cytokines containing IL/1 (Interleukin 1) and TNF-α. In a similar experimental study, gene expressions of IL-1β (Interleukin 1 beta) and TNF-α increased in ovarian tissue due to ischemia/reperfusion damage^38^. In our study, the TNF-α expression was increased in antral follicular cells, inflammatory cells, and endothelial cells due to cell degeneration from increased inflammation in the ischemia and ischemia-reperfusion groups. In the ischemia-reperfusion with RA group, a decreased degenerative effect and inflammation-induced TNF-α expression were positive in some macrophage cells away from preantral and antral follicles, whereas TNF-α expression was negative in other areas.

Caspase-3 is a regulator of the apoptotic enzyme cascade. Sapmaz-Metin et al.^39^ observed that the apoptotic cell number increased significantly in the ovaries after ischemia/reperfusion. They detected TUNEL positive granulosa cells only in medium or large ovarian follicles. They reported that an ischemia/reperfusion injury does not reduce the ovarian germ cell pool but instead leads to oocyte maturation problems due to the loss of some internal factors mediated by granulosa cell death. In the ischemia group in our study, the activation of caspase-3 was observed in degenerated granule cells of preantral and antral follicles, luteal cells of the corpus luteum, and many inflammatory cells in the stromal region (Fig. 4b). In the ischemia-reperfusion group, a significant increase in caspase-3 activation was observed in the granular cells in mature antral follicles and in inflammatory cells in the stromal region (Fig. 4c). In the ischemia-reperfusion with RA group, caspase-3 expression was negative in the antral follicle cells and granular cells around the antral follicle (Fig. 4d).

The duration and severity of ischemia and the prolongation of the reperfusion time play an important role in the development of damage to the cells due to this reaction. It is observed that RA, which is applied after an ischemia/reperfusion injury, increases the vascularization in the follicle cells and luteal cells while decreasing the endothelial cells. Ischemia is an indicator of increased degeneration damage during the development of cytokine activation with increased inflammation after ischemia/reperfusion. In addition, RA after ischemia/reperfusion provided positive protection against inflammatory cell infiltration and reduced the degenerative effect. Apoptotic cells increased in the ischemia and ischemia-reperfusion groups in preantral and antral follicle cells, and luteal cells decreased after RA administration.

## Conclusions

Rosmarinic acid application has shown to have an antioxidative effect by decreasing the increased lipid peroxidation after ischemia, and it has a positive effect in preventing tissue damage by reducing super oxide dysmutase. It is thought that increased VEGF activity in endothelial cells induces angiogenetic development, stimulates TNF-α signal pathway and regulates inflammation, and also plays an important role in preventing apoptosis by decreasing Caspase-3 activity. Therefore, we suggest that rosmarinic acid given at a dose of 50 mg/kg may be useful in preventing ischemic damage after vascularization, inflammation, and apoptotic development after ischemia/reperfusion.

## Footnotes

1 Research performed at Experimental Research Center, and Histology and Embryology Laboratory, Dicle University, Diyarbakır, Turkey.
